# Estimating the Proportion of *Plasmodium vivax* Recurrences Caused by Relapse: A Systematic Review and Meta-Analysis

**DOI:** 10.4269/ajtmh.20-0186

**Published:** 2020-06-08

**Authors:** Robert J. Commons, Julie A. Simpson, James Watson, Nicholas J. White, Ric N. Price

**Affiliations:** 1 Global Health Division, Menzies School of Health Research and Charles Darwin University, Darwin, Australia;; 2 Internal Medical Services, Ballarat Health Services, Ballarat, Australia;; 3 Centre for Epidemiology and Biostatistics, Melbourne School of Population and Global Health, The University of Melbourne, Melbourne, Australia;; 4 Mahidol-Oxford Tropical Medicine Research Unit (MORU), Faculty of Tropical Medicine, Mahidol University, Bangkok, Thailand;; 5 Centre for Tropical Medicine and Global Health, Nuffield Department of Clinical Medicine, University of Oxford, Oxford, United Kingdom

## Abstract

*Plasmodium vivax* and *Plasmodium ovale* form dormant liver hypnozoites that can reactivate weeks to months following initial infection. Malaria recurrences caused by relapses are an important cause of morbidity and source of transmission. To estimate the proportions of *P. vivax* malaria recurrences caused by relapses in different geographical locations, we systematically reviewed clinical efficacy studies of uncomplicated *P. vivax* malaria, in which patients were randomized to treatment with or without radical cure primaquine regimens and were followed up for 1 year. The minimum proportion of recurrences caused by relapses was estimated for each study site by assuming primaquine prevented all relapses and did not augment blood-stage efficacy. Of the 261 studies identified, six were eligible enrolling 4,092 patients from 14 treatment arm comparisons across seven countries. Of the 2,735 patients treated with primaquine, 24.3% received low dose (2.5 to < 5.0 mg/kg total) and 75.7% received high-dose primaquine (≥ 5.0 mg/kg total). The overall pooled incidence rate ratio of *P. vivax* relapses for patients treated with primaquine versus no primaquine was 0.15 (95% CI: 0.10–0.21; *I*^*2*^ = 83.3%), equating to a minimum of 79% of recurrences attributable to relapse. Country-specific incidence rate ratios ranged from 0.05 (95% CI: 0.01–0.34; one estimate) in Pakistan to 0.34 in Nepal (95% CI: 0.12–0.83; one estimate) and Afghanistan (95% CI: 0.22–0.51; three estimates). Relapses account for a very high proportion of recurrent infections following schizontocidal treatment of acute *P. vivax* malaria across diverse geographic locations. This emphasizes the importance of implementing hypnozoitocidal treatment.

## INTRODUCTION

*Plasmodium vivax* malaria caused an estimated 14.3 million infections in 2017.^1^ Unlike *Plasmodium falciparum*, successful treatment of blood-stage infection does not necessarily prevent recurrence. Dormant liver hypnozoites can reactivate weeks to months after the initial infection and cause recurrent parasitemia (relapse) and further episodes of clinical malaria. Recurrent malaria can also be caused either by failure to clear blood-stage infection effectively (recrudescence) or by a new infection following exposure to another infected mosquito (reinfection).^2^ Effective treatment of *P. vivax* malaria requires treatment of both the blood stage and persistent liver stages of the parasite; this is termed radical cure. Primaquine is the only widely available antimalarial for the treatment of dormant liver-stage parasites, which reduces the risk of relapse by over 90%, with higher doses having greater anti-relapse efficacy.^3,4^ Because relapsing infections are important drivers of transmission of *P. vivax* malaria,^5^ quantification of the proportion of recurrences caused by relapse is critical to our understanding of the potential benefits of radical cure in different endemic locations.

Identifying the cause of *P. vivax* recurrence is complex. Molecular analysis of the pre- and posttreatment parasites is less informative than that in *P. falciparum* infections because relapses can be genetically similar to or different from the initial infection.^6,7^ Data from two clinical studies have been used to estimate the proportion of *P. vivax* parasitemias attributable to relapse in populations in Southeast Asia. A study in children aged 5–10 years in Papua New Guinea (PNG) conducted in 2009–2010 compared asymptomatic patients treated with and without prolonged high-dose primaquine and estimated that 82% of *P. vivax* episodes in this high-transmission setting resulted from relapses.^8^ A complementary analysis of a cohort from the Thailand–Myanmar border and a separate cohort from PNG estimated relapses to cause 70% and 96% of recurrences, respectively, when assuming the anti-relapse preventive effectiveness of primaquine was 100%.^9^ Transmission intensities, seasonality, and the relapse periodicity of *P. vivax* vary considerably between locations.^10^ Thus, the proportion of recurrences attributable to *P. vivax* relapses will differ by region, location, patients’ age, and season. Nevertheless, extrapolation of the benefits of radical cure in reducing *P. vivax*–associated morbidity, mortality, and transmission across the *P. vivax*–endemic world requires some estimate. Using data from anti-relapse efficacy studies, a number of which were published recently, this study aimed at providing an estimate of the proportion of *P. vivax* recurrences due to relapses in locations with different malarial endemicity.

## METHODS

### Data extraction.

Medline, Embase, Web of Science, and the Cochrane Database of Systematic Reviews were searched for prospective clinical efficacy studies of uncomplicated *P. vivax* malaria, as described previously.^3^ Studies published before August 6, 2019 in any language were included if they included patients with *P. vivax* malaria treated with and without primaquine, provided that a minimum total dose of primaquine of 2.5 mg base/kg was commenced within 3 days of enrollment and administered daily thereafter in one treatment arm and that there was active follow-up for 365 days through multiple episodes of *P. vivax* parasitemia should they occur. Studies were excluded if they were retrospective, patients were only followed up passively, or incidence rates could not be extracted. Studies were identified by two authors (R. J. C. and R. N. P.) using the search terms in Supplemental File 1, with discrepancies resolved by discussion.

Data were extracted for each treatment site and treatment arm. When multiple primaquine regimens were used within a study, data were aggregated by total mg/kg primaquine dose irrespective of the number of days over which the primaquine regimen was given. Extracted data included details of the study, site, duration of follow-up, schizontocidal treatment, primaquine dose and duration, patient numbers, and incidence rate of parasite recurrence over 365 days. Investigators of eligible studies in which incidence rates could not be extracted from the published article were contacted directly. Primaquine dose was defined as low if the target total dose of primaquine was 2.5–<5.0 mg/kg and high if ≥ 5.0 mg/kg.^4^

### Analysis.

The primary outcome of the analysis was the incidence rate of *P. vivax* recurrences over 365 days following treatment for each study site, as reported in the article. The incidence rate ratio was calculated for each study site using the ratio of the incidence rate of *P. vivax* recurrences in the treatment arm with primaquine versus the treatment arm without primaquine. The minimum proportion of recurrences attributable to relapse was estimated by subtracting the incidence rate ratio from 1. These estimates make two key assumptions: first, primaquine does not significantly improve the asexual parasite killing effect and, thus, the cure rate of the antimalarial treatment, and second, the treatment arms with primaquine were 100% effective in preventing relapses. A follow-up duration of 365 days was chosen to ensure that relapses from long-latency *P. vivax* were captured.

The pooled incidence rate ratio of recurrent *P. vivax* parasitemia in patients treated with primaquine versus those without was estimated using a Poisson regression model with random study effects.^11,12^ A random-effects model was used because the incidence rate ratio varies between geographic locations and studies. Subgroup estimates were calculated for primaquine dose and country. Between-study heterogeneity was quantified by the *I*^*2*^ statistic. Analyses were performed using Stata version 15 (StataCorp, College Station, TX) and R version 3·4·0 (R Foundation for Statistical Computing, Vienna, Austria). The study protocol was registered with PROSPERO: CRD42020158857.

## RESULTS

**Figure 1.**
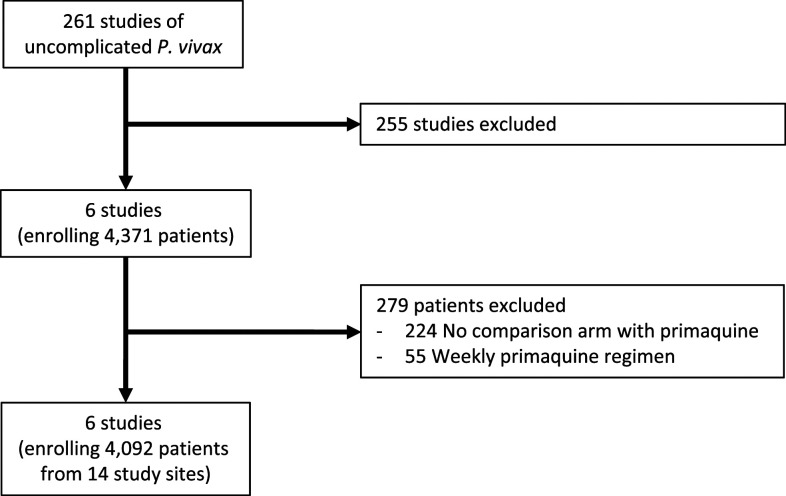
Flow diagram. Reasons for study exclusion are detailed in Supplemental File 2.

The systematic review identified 261 studies of uncomplicated *P. vivax* malaria treatment, of which 39 (14.9%) recruited and followed up patients for 365 days (Figure 1, Supplemental File 2). Of these, 33 studies were excluded: 30 studies did not compare the same treatment with or without primaquine, one study gave weekly primaquine, and in two studies, the total primaquine dose was < 2.5 mg/kg (Supplemental File 2). Of the remaining six studies, 224 patients from one study were excluded from an artesunate treatment arm for which there was no comparative arm with primaquine^13^ and 55 patients from one study were excluded from a treatment arm because of treatment with weekly primaquine.^14^ In total, 4,092 patients were included in the analysis from six studies^13–18^ across 14 paired treatment arms with and without primaquine (Table 1). Data to calculate incidence rates were extracted from four studies directly, with additional data on incidence rates provided by investigators for two studies.

**Table 1:** Included study details and outcomes

Study	Site	Country	Years enrolled	Randomized	Blinded	Age range (years)	Treatment	PQ dose category	PQ regimen	PQ supervision	No. without PQ	No. with PQ	Recurrences without PQ	Person months without PQ	Recurrences with PQ	Person months with PQ	Incidence rate ratio	Recurrences caused by relapse (%)
Leslie et al.^14^	Peshawar	Pakistan	2004–2006	Yes	No	16–88	Chloroquine	Low	0.25 × 14 = 3.5 mg/kg	Full	71	74	22	589	1	588	0.05	95
Abreha et al.^16,^†	Oromia region	Ethiopia	2012–2014	Yes	No	1–67	Chloroquine	Low	0.25 × 14 = 3.5 mg/kg	Partial (5 days)	104	102	133	725.4	26	780	0.19	81
Abreha et al.^16,^*	Oromia region	Ethiopia	2012–2014	Yes	No	1–65	AL	Low	0.25 × 14 = 3.5 mg/kg	Partial (5 days)	100	92	134	699.12	29	696	0.22	78
Awab et al.^15^	Jalalabad†	Afghanistan	2009–2013	Yes	No	2–84	Chloroquine	Low	0.25 × 14 = 3.5 mg/kg	Partial (4 days)	295	292	105	3,187.64	41	3,192	0.39	61
Rijal et al.^18^	Kailali†	Nepal	2015–2017	Yes	No	5–75	Chloroquine	Low	0.25 × 14 = 3.5 mg/kg	Partial (5 days)	101	105	22	973.3	7	905.4	0.34	66
Chu et al.^13^	Shoklo	Thai-Myanmar border	2010–2012	Yes	No	1–63	Chloroquine	High	0.5 × 14 = 7 mg/kg	Full	222	198	587	2,004	40	1,848	0.08	92
Taylor et al.^17,^‡	Jalalabad	Afghanistan	2014–2017	Yes	Yes	0–70	Chloroquine	High	1 × 7 or 0.5 × 14 = 7 mg/kg	Full	60	251	17	208.88	17	1,125.12	0.19	81
Taylor et al.^17,^‡	Laghman	Afghanistan	2014–2017	Yes	Yes	0–70	Chloroquine	High	1 × 7 or 0.5 × 14 = 7 mg/kg	Full	23	97	14	109.37	30	693.42	0.34	66
Taylor et al.^17,^‡	Arba Minch	Ethiopia	2014–2017	Yes	Yes	1–70	Chloroquine	High	1 × 7 or 0.5 × 14 = 7 mg/kg	Full	74	297	42	260.91	34	1,841.13	0.11	89
Taylor et al.^17,^‡	Metehara	Ethiopia	2014–2017	Yes	Yes	2–54	Chloroquine	High	1 × 7 or 0.5 × 14 = 7 mg/kg	Full	40	169	22	180.31	10	1,244.94	0.07	93
Taylor et al.^17,^‡	Hanura	Indonesia	2014–2017	Yes	Yes	0–70	DP	High	1 × 7 or 0.5 × 14 = 7 mg/kg	Full	118	457	59	879.55	62	4,462.88	0.21	79
Taylor et al.^17,^‡	Tanjung Leidong	Indonesia	2014–2017	Yes	Yes	1–67	DP	High	1 × 7 or 0.5 × 14 = 7 mg/kg	Full	85	340	21	662.43	15	3,015.55	0.16	84
Taylor et al.^17,^‡	Dak O & Bu Gia Map	Vietnam	2014–2017	Yes	Yes	3–58	Chloroquine	High	1 × 7 or 0.5 × 14 = 7 mg/kg	Full	43	176	31	166.96	17	1,303.48	0.07	93
Taylor et al.^17,^‡	Krong Pa	Vietnam	2014–2017	Yes	Yes	10–94	Chloroquine	High	1 × 7 or 0.5 × 14 = 7 mg/kg	Full	21	85	9	111.11	1	862.69	0.01	99

AL = artemether–lumefantrine; API = annual parasite index (per 1,000 per year); DP = dihydroartemisinin–piperaquine; PQ = primaquine; U = unknown.

* Patients initially treated with supervised primaquine received unsupervised primaquine on presentation with recurrence.

† Patients enrolled from multiple sites, but only aggregated data available.

‡ Primaquine arm includes patients treated with 7 mg/kg over 7 days and 14 days.

Overall, 1,018 patients were enrolled in Afghanistan (three paired treatment arms), 978 in Ethiopia (four paired treatment arms), 1,000 in Indonesia (two paired treatment arms), 420 from the Thailand–Myanmar border (one paired treatment arm), 325 in Vietnam (two paired treatment arms), 206 in Nepal (one paired treatment arm), and 145 in Pakistan (one paired treatment arm). The blood schizonticide administered was chloroquine in 2,983 (72.9%) patients, dihydroartemisinin–piperaquine in 1,000 (24.4%) patients, and artemether–lumefantrine in 192 (4.7%) patients. A low-dose primaquine regimen (0.25 mg/kg for 14 days) was given to 665 (24.3%) patients from five treatment arms, and a high-dose regimen (0.5 mg/kg for 14 days or 1 mg/kg for 7 days) was given to 2,070 (75.7%) patients from nine treatment arms.

**Figure 2.**
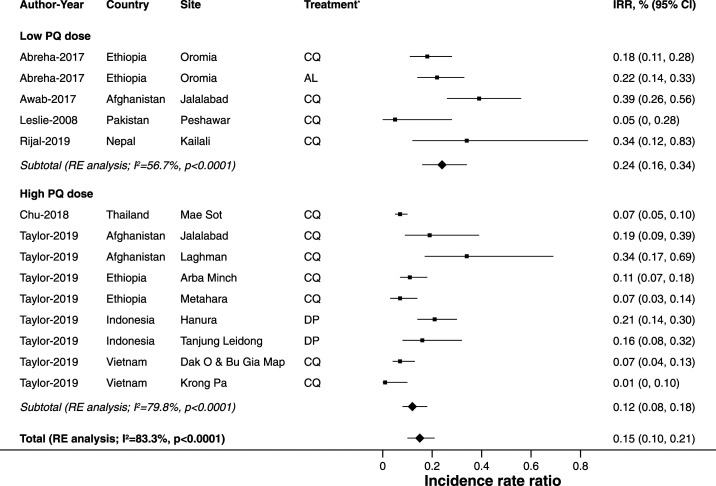
Pooled estimates of the incidence rate ratios of *P. vivax* recurrences within 365 days of follow-up comparing treatment with primaquine with no primaquine by total primaquine dose administered (low dose 2.5 to < 5 mg/kg, high dose ≥ 5 mg/kg). AL = artemether–lumefantrine; CQ = chloroquine; DP = dihydroartemisinin–piperaquine; IRR = incidence rate ratio. * Blood schizontocidal treatment.

Compared with patients who were not treated with primaquine, the incidence rate ratios of *P. vivax* recurrences for patients treated with primaquine ranged from 0.05 to 0.39, with an overall pooled incidence rate ratio of 0.15 (95% CI: 0.10–0.21; *I*^*2*^ = 83.3%; 14 estimates; Figure 2), consistent with 85% of recurrences being due to relapses, or conservatively 79% based on the upper CI.

**Figure 3.**
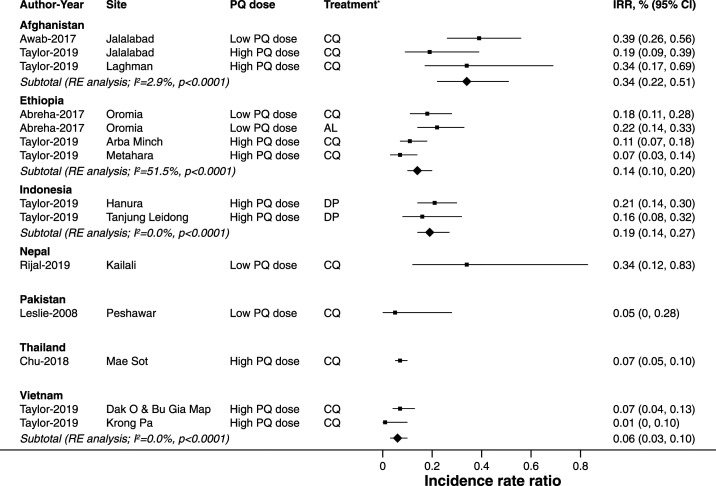
Pooled estimates of incidence rate ratios of *P. vivax* recurrences within 365 days of follow-up comparing treatment with primaquine with no primaquine by country. AL = artemether–lumefantrine; CQ = chloroquine; DP = dihydroartemisinin–piperaquine; IRR = incidence rate ratio *Blood schizontocidal treatment.

Patients treated with a low dose of primaquine had a pooled incidence rate ratio of 0.24 (95% CI: 0.16–0.34; *I*^*2*^ = 56.7%; five estimates), and patients treated with a high dose of primaquine had a pooled incidence rate ratio of 0.12 (95% CI: 0.08–0.18; *I*^*2*^ = 79.8%; nine estimates). Country-specific estimates ranged from 0.05 (95% CI: 0.01–0.34; one estimate) in Pakistan to 0.34 in Nepal (95% CI: 0.12–0.83; one estimate) and also 0.34 in Afghanistan (95% CI: 0.22–0.51; three estimates), suggesting a minimum of 66% of recurrences were caused by relapse (Figure 3).

Methodological factors potentially contributing to bias are presented in Table 1. Most studies re-treated patients with recurrence with the same treatment that they were initially allocated. However, in one study, patients with recurrence were re-treated with unsupervised primaquine, after initial treatment with supervised primaquine.^16^ Because this will have potentially led to attenuation of the estimated incidence rate ratio, the analysis was repeated excluding this study. The overall pooled incidence rate ratio of 0.14 (95% CI: 0.09–0.21; *I*^*2*^ = 84.8%; 12 estimates) was consistent with 86% of recurrences resulting from relapses.

## DISCUSSION

In this meta-analysis, the minimum proportion of *P. vivax* recurrences related to relapse was estimated across several *P. vivax*–endemic countries from data derived from anti-relapse clinical trials. Under the reasonable assumption that the only effect of primaquine was to eliminate hypnozoites, overall, 85% of recurrences were estimated to be related to relapses, with a minimum country-based estimate of 66% recorded in Afghanistan and Nepal. The study highlights a consistently high burden of *P. vivax* episodes attributable to relapse across diverse endemic settings. Previous estimates are based on clinical trials conducted in Southeast Asia and are thus potentially biased by the high relapse rates of *P. vivax* malaria in these areas and likely high hypnozoite burdens.^1,10^

The high proportion of recurrences caused by relapse in our study supports a recent population model of recurrent parasitemia on the Thailand-Myanmar border, in which the cause of recurrent infections was estimated probabilistically by incorporating molecular information on genetic relatedness and time to recurrence.^19^ In this location, 95% of recurrent *P. vivax* episodes following treatment with chloroquine were attributed to relapse.

We estimated the minimum proportion of recurrent *P. vivax* episodes related to relapse within a year of an initial infection, without ascribing the origin of the initial infection as being a relapse or a new infection. This differs from the earlier study by Robinson et al.^8^ which estimated the proportion of all *P. vivax* episodes within a population caused by relapse by following up a cohort of patients after treatment with a very high dose of primaquine (10 mg/kg total dose) to clear residual hypnozoites.

To derive a lower bound on the proportion of recurrence due to relapse, we assumed that primaquine treatment was 100% effective and, thus, that any recurrences following primaquine treatment resulted from background reinfection. In reality, this is not the case, as highlighted by clinical trials in which patients are not re-exposed to reinfection, receive a full course of radical treatment, and yet continue to have recurrent infections.^20,21^ Relapses following primaquine treatment are related to host, parasite, and drug factors. Host factors include treatment adherence, innate and acquired immunity, and cytochrome P450 2D6 (CYP2D6) polymorphisms. Drug factors include the dose of primaquine and pharmacokinetic–pharmacodynamic interactions.^22,23^ Parasite factors include hypnozoite burden, latency, and intrinsic drug susceptibility.^10^ Hence, the optimal dose for primaquine may vary markedly between different endemic regions.^24^ The comparative benefit of low- and high-dose primaquine regimens and how this differs across different endemic settings remains unclear. As expected, our analysis found that the pooled incidence rate ratios were lower following high-dose primaquine (0.12) than following low-dose primaquine (0.24), consistent with anti-relapse efficacy being related to primaquine dose. In both cases, our estimates of the proportion attributable to relapses are conservative, as our assumption of 100% primaquine anti-relapse efficacy will lead to an underestimation of the relapse-attributable proportion of recurrences.

Host CYP2D6 loss of function polymorphisms are associated with reduced primaquine efficacy,^23^ and their prevalence also differs between populations.^25^ Similar to the underdosing of primaquine, inclusion of patients with reduced primaquine metabolism will have led to reduced efficacy. Thus, our assumption of 100% primaquine anti-relapse efficacy will not have been correct for patients with some CYP2D6 polymorphisms, leading us to further underestimate the relapse-attributable proportion of recurrences.

The known geographic differences between CYP2D6 polymorphisms and primaquine anti-relapse efficacy, combined with differences in transmission over time, may have contributed to the identified heterogeneity between study sites in the current study. In addition, variations in supervision of primaquine dosing and differing durations of primaquine treatment will lead to variations in adherence and, thus, total primaquine dose administered.

The use of primaquine treatment as the primary comparator arm also assumes that the risk of recrudescence is similar in patients treated with and without primaquine. However, primaquine has blood schizontocidal activity that reduces early recurrent infection likely from both recrudescence and relapse.^26,27^ In locations with reduced chloroquine susceptibility, the number of recrudescences may have been lower in the primaquine treatment arms, resulting in an overestimation of the proportion of recurrences attributable to relapse. However, this is likely to have had a minimal impact on the analysis because the Indonesian study, the only one in this analysis with highly chloroquine-resistant *P. vivax*, used dihydroartemisinin–piperaquine as the blood schizontocide.

Chloroquine and piperaquine have long half-lives that may suppress the first relapse,^13^ and this may have reduced the number of recurrent infections in the treatment arm without primaquine, thus underestimating the proportion of recurrences attributable to relapse.

Restricting the inclusion criteria to studies with 365 days of follow-up ensured that relapses from long-latency *P. vivax* were detected and thereby enabled direct comparisons of incidence rates. However, limiting study eligibility risks selection bias and a reduction in the generalizability of our analysis.

In summary, in all studies included in this analysis, a very high proportion of *P. vivax* malaria recurrences were attributable to relapse, and this was consistent across a diverse range of *P. vivax*–endemic settings. As progress toward malaria elimination begins to falter,^28^ our study once again highlights that hypnozoites are the single most important target to change the current trajectory and reduce the *P. vivax* prevalence. Recent advances in glucose-6-phosphate dehydrogenase (G6PD) diagnostics^29^ and novel regimens for radical cure such as high-dose short-course primaquine^17,30^ and single-dose tafenoquine^31,32^ offer important alternative approaches for the radical cure of *P. vivax* malaria. The high proportion of recurrences attributable to relapse suggests that if implemented safely and effectively, improved strategies to ensure radical cure would have a significant public health impact. This will be critical in achieving the ambitious malaria elimination targets in the Asia-Pacific, Americas, and Horn of Africa.

## Supplemental files

Supplemental materials
