# Supplementary material for: Estimating the Proportion of Plasmodium vivax Recurrences Caused by Relapse: A Systematic Review and Meta-Analysis
Source: Am J Trop Med Hyg. 2020 Jun 8;103(3):1094–9. doi: 10.4269/ajtmh.20-0186 (PMC7470578; doi:10.4269/ajtmh.20-0186)
Supplement: Supplementary file 2 [file tpmd200186.SD2.docx]

**Supplementary File 1. Search strategy**

Literature search (conducted August 2019) with the following key terms (version undertaken in Pubmed):

*vivax* AND (*amodiaquine* OR *atovaquone* OR *artemisinin* OR *arteether* OR *artesunate* OR *artemether* OR *artemether* OR *artemotil* OR *azithromycin* OR *artekin* OR *chloroquine* OR *chlorproguanil* OR *cycloguanil* OR *clindamycin* OR *coartem* OR *dapsone* OR *dihydroartemisinin* OR *duo-cotecxin* OR *doxycycline* OR *halofantrine* OR *lumefantrine* OR *lariam* OR *malarone* OR *mefloquine* OR *naphthoquine* OR *naphthoquinone* OR *piperaquine* OR *primaquine* OR *proguanil* OR *pyrimethamine* OR *pyronaridine* OR *proguanil* OR *quinidine* OR *quinine* OR *riamet* OR *sulphadoxine* OR *tetracycline*OR *tafenoquine*).

**Supplementary File 2. Systematic review**

*Note:* Supplemental File 2 will be available online in final publication
